# Windowed Correlation: A Suitable Tool for Providing Dynamic fMRI-Based Functional Connectivity Neurofeedback on Task Difficulty

**DOI:** 10.1371/journal.pone.0085929

**Published:** 2014-01-20

**Authors:** Anna Zilverstand, Bettina Sorger, Jan Zimmermann, Amanda Kaas, Rainer Goebel

**Affiliations:** 1 Department of Cognitive Neuroscience, Faculty of Psychology and Neuroscience, Maastricht University, Maastricht, The Netherlands; 2 Department of Neuroimaging and Neuromodeling, Netherlands Institute for Neuroscience, Royal Netherlands Academy of Arts and Sciences, Amsterdam, The Netherlands; University of Minnesota, United States of America

## Abstract

The goal of neurofeedback training is to provide participants with relevant information on their ongoing brain processes in order to enable them to change these processes in a meaningful way. Under the assumption of an intrinsic brain-behavior link, neurofeedback can be a tool to guide a participant towards a desired behavioral state, such as a healthier state in the case of patients. Current research in clinical neuroscience regarding the most robust indicators of pathological brain processes in psychiatric and neurological disorders indicates that fMRI-based functional connectivity measures may be among the most important biomarkers of disease. The present study therefore investigated the general potential of providing fMRI neurofeedback based on functional correlations, computed from short-window time course data at the level of single task periods. The ability to detect subtle changes in task performance with block-wise functional connectivity measures was evaluated based on imaging data from healthy participants performing a simple motor task, which was systematically varied along two task dimensions representing two different aspects of task difficulty. The results demonstrate that fMRI-based functional connectivity measures may provide a better indicator for an increase in overall (motor) task difficulty than activation level-based measures. Windowed functional correlations thus seem to provide relevant and unique information regarding ongoing brain processes, which is not captured equally well by standard activation level-based neurofeedback measures. Functional connectivity markers, therefore, may indeed provide a valuable tool to enhance and monitor learning within an fMRI neurofeedback setup.

## Introduction

In neurofeedback training, participants are provided with online feedback on their current individual brain processes. The rationale is that feedback on current brain processing may provide a useful tool for guiding participants towards a desired behavioral state, if the tapped brain correlates are intrinsically linked to relevant phenomena on the behavioral, cognitive and emotional level. Neurofeedback training may thus provide a method for changing brain activation and alleviating symptoms in patients with pathological brain patterns. Preliminary evidence supports the idea that neurofeedback training interventions based on functional magnetic resonance imaging (fMRI) can induce specific changes in behavior, emotion and cognition in healthy participants as well as in patients with psychiatric and neurological disorders [1], [2], [3]. Importantly, previous research demonstrates that effective fMRI-based neurofeedback training is dependent on feeding back the information most relevant for the desired change, for example, giving feedback from brain regions, which are modulated by task performance [2]. However, up to now, different measures derived from fMRI data have not been systematically compared regarding their suitability to provide the most useful or effective feedback. As a first step towards answering this question, the present study aims at investigating whether fMRI-based functional connectivity and activation-level based measures provide the same or different information regarding relevant aspects of different versions of a simple motor task in healthy participants.

The motivation for this study stems from the increased interest in using functional connectivity analysis for investigating biological markers of psychiatric and neurological disorders. It has been claimed that brain connectivity biomarkers are among the most robust indicators of clinical disorders [4]. Deviant functional connectivity patterns in patients have been linked to behavioral, cognitive and emotional symptoms in disorders as diverse as attention deficit hyperactivity disorder [5], schizophrenia [6], autism [7], anxiety [8], mood disorders [9], and movement disorders [10]. For example, research on treatment effects has led to the hypothesis that the most prominent change after successful pharmacological treatment in attention deficit hyperactivity disorder may be the normalization of abnormal task-relevant functional connectivity patterns, while localized changes of brain activation level seemed to be less indicative [11]. Similarly, a link between a positive treatment response to antidepressant medication and the normalization of cingulate-amygdala connectivity has been drawn [12], [13]. Consequently, the increasing focus on the importance of dysfunctional connectivity networks in psychiatric and neurological diseases during the last decade has also lead to an increased interest in using functional connectivity measures as an fMRI-based neurofeedback signal [1], [2], [14], [15], [16]. A recent study on the feasibility of using effective connectivity measures within a neurofeedback setup showed that participants are indeed able to voluntarily control such a feedback signal [17]. However, to the best of our knowledge, no systematic comparison regarding the potential of using functional connectivity versus activation level-based measures as a feedback signal in the context of neurofeedback training has been conducted so far.

Functional brain networks emerge when local brain regions interact in order to integrate different task aspects. Two concepts to measure this interaction have been defined in fMRI research: the concept of ‘effective connectivity’, which aims at measuring how much “influence one neural system exerts over another”, and the concept of ‘functional connectivity’: the mere “statistical dependency among neurophysiological events” [18]. Windowed fMRI correlation measures as implemented here estimate functional connectivity by measuring the amount of common variance in the activation-level changes of two or more circumscribed brain regions during a short time interval. While functional connectivity measures are essentially data-descriptive and not a direct measurement of the underlying neural interactions [18], they have been utilized as an indicator for the ongoing integration on the neuronal level during task performance [19]. Different types of connectivity measures have been categorized into more simple, model-free methods (e.g., correlation) versus more complex modeling methods (e.g., dynamic causal modelling), which may provide more meaningful information (e.g., on the directionality of neural processes), but are computationally more expensive [20]. For real-time data analysis, the feedback signal needs to be computed within relatively short time windows, thus a method providing robust estimates with a few data points may be advantageous in this context. The modeling approach implemented by Koush and colleagues (2013), a dynamic causal modelling approach, required a sliding window with a length of 90 seconds for stable model estimation while statistically stable correlations can be computed based on shorter time windows. Importantly, a systematic comparison regarding the sensitivity of different connectivity methods showed that the correlation method has good sensitivity, performing among the top four of twelve investigated methods [21]. Another common division of functional connectivity methods is the separation between hypothesis-driven seed-based methods using a priori-selected regions of interest and data-driven methods that partition the data into functional networks based on statistical criteria, as for example independent component analysis. A systematic comparison between these two sorts of methods using simulations and offline analysis of imaging data showed considerable convergence in the results, both regarding the spatial layout of the functional networks as well as regarding the estimation of connectivity strength [22]. While both types of methods have been made available for real-time data analysis (e.g., sliding window correlation analysis, [23]; sliding window independent component analysis, [24]; single trial-based multi-filter correlation analysis [25]), the hypothesis-driven seed-based methods seem more suitable when the aim is to achieve high spatial specificity within short-time windows. High dimensional independent component analysis, for example, which partitions the data into highly spatially differentiated components, also requires rather long sliding windows. We therefore investigated the potential of using a hypothesis-driven, computationally inexpensive method: windowed seed-based correlation. An important characteristic of this method is that correlation measures have been shown to be susceptible to the influence of noise artifacts [18], [20]. We therefore employed noise regression, a method which is suitable for real-time data analysis, to remove common noise artifacts [26], [27], [28], [29], [30], [31].

**Figure 1:**
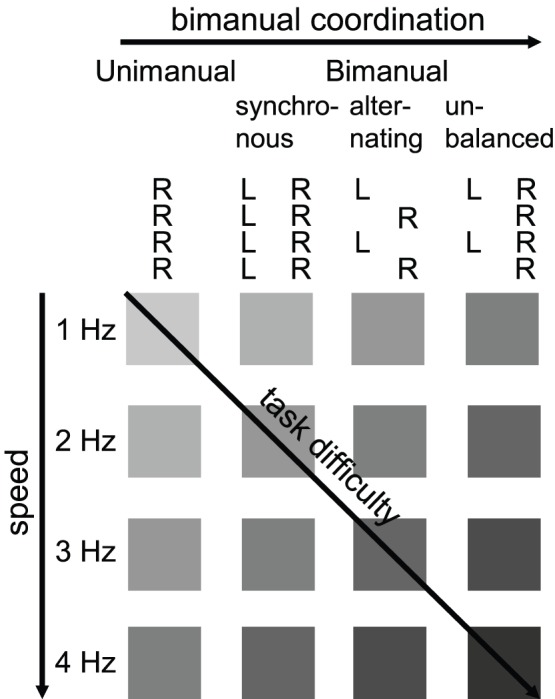
Experimental Design. The participants performed four different types of tapping sequences, which were selected to increase demand on bimanual coordination gradually: 1) *unimanual*: moving only the right index finger, 2) *bimanual synchronous*: moving both index fingers in synchrony, 3) *bimanual alternating*: moving both index fingers at the same pace in an alternating fashion, and 4) *bimanual unbalanced*: moving the left index finger in synchrony with the right index finger, but at half of the pace. Each of these tapping sequences was performed at four different tapping speeds for the right index finger (1, 2, 3, and 4 Hz), which resulted in 16 different experimental conditions. Task difficulty increased along both manipulated task dimensions.

In order to investigate how well functional connectivity and activation level-based measures may serve as an indicator of subtle changes in task performance, we asked healthy participants to perform a simple finger tapping task that was systematically modulated along two dimensions, namely tapping speed and demand on bimanual coordination, thus combining two aspects of task difficulty (see **figure 1**). The implementation of the selected task allowed computing individual block-wise performance measures based on both activation levels and functional connectivity, in order to compute brain-behavior correlations. Finger tapping tasks have been well studied regarding their associated effects on functional connectivity, and their effects on the activation level. It has long been known that the activation level within the whole motor network increases with increasing finger tapping speed [32], [33], and there is some evidence that increasing demand on bimanual coordination raises activation level as well [34]. Furthermore, the first study on the effects of finger tapping on functional connectivity showed that the correlation between voxels within the motor network was higher during task performance than rest [35]. This result has been replicated several times (e.g. [36], [37], [38], [39]), and extended to show that the functional connectivity between the motor and visual network was higher during a visuo-motor task [40]. Also, a learning experiment on finger tapping [41] has shown that functional connectivity increased during early, as compared to later, learning stages, thus being modulated by general task difficulty. Two other studies provided evidence for a parametric modulation of the correlation strength by finger tapping speed within the motor network [37], and found that differing degrees of demand on bimanual coordination influenced the strength of the functional connection between the bilateral motor regions [36]. Interestingly, they concluded (but did not test) that functional connectivity measures may serve as a better indicator regarding the demand on bimanual coordination than the activation-level based measures. While several studies have thus shown task-dependent modulations of functional connectivity as well as of activation level, none of these studies reported individual brain-behavior correlations that would be needed to evaluate its suitability for real-time analysis. In order to investigate how sensitive and specific these brain activation measures are for detecting the chosen task manipulations in short time windows, we combined, replicated and extended the previous studies by including several different bimanual task variations. All brain measures were derived from short-window time-course data on single-trial level. We hypothesized that while activation-level measures might be a stronger correlate of the individually performed tapping speed, functional connectivity measures might be more sensitive in detecting the demand on bimanual coordination.

## Methods

### Participants and Ethics Statement

Five healthy volunteers participated in the study (mean age: 29.4±2.8 years). All participants were right-handed as evaluated by the Edinburgh Handedness Inventory [42]. Participants gave their written informed consent prior to the experiment that was conducted in conformity with the Declaration of Helsinki and approved by the local Ethics Committee of the Faculty of Psychology and Neuroscience at Maastricht University.

### Experimental Procedure

Before the scanning session, participants were trained for half an hour outside the scanner to get familiarized with the motor task. During the MRI session (approx. 2 h) a block design with 20-s task blocks and alternating 20-s rest periods was employed. During task blocks participants were guided by a visual metronome that consisted of a flickering ‘R’ on the right side (indicating right index finger pace) and a flickering ‘L’ on the left side (indicating left index finger pace). This display was generated using the Presentation software package (Version 16, Neurobehavioral Systems Inc., Albany, CA, USA), and was projected onto a mirror mounted in the scanner in front of the participant’s head. During the task blocks of a localization run (11 min) participants were instructed to tap at a medium speed of 2 Hz with their left index finger or their right index finger only. During the following eight experimental runs (11 min each) the participants performed four different types of tapping sequences, which required an increasing demand on bimanual coordination: 1) moving only the right index finger (*unimanual*), 2) moving both index fingers in synchrony (*bimanual synchronous*), 3) moving both index fingers at the same pace in an alternating fashion (*bimanual alternating*), and 4) moving the left index finger in synchrony with the right index finger, but at half of the pace (*bimanual unbalanced*). Each of these tapping sequences was performed at four different right-finger tapping speeds (1, 2, 3, and 4 Hz), which resulted in 16 different experimental conditions (**figure 1**). Eight repetitions per condition were implemented for each participant (total of 128 task blocks), with the order being counterbalanced across the session. All finger movements during tapping were recorded using a button box, and the software Presentation. The imaging session concluded with the acquisition of the anatomical images.

### MRI Data Acquisition

The images were acquired at Maastricht Brain Imaging Centre (Maastricht University) on a 3T scanner, (Magnetom Allegra, Siemens Healthcare, Germany), equipped with a standard quadrature birdcage head coil. The participants were placed comfortably in the scanner and their heads were fixed with foam cushions to minimize task-related and other spontaneous motion. All participants were instructed to avoid any movement other than the finger tapping during scanning. Functional images were acquired with a repeated single-shot echo-planar imaging (EPI) sequence with a relatively short repetition time (TR = 1000 ms), adjusted flip angle (FA = 62°), standard echo time (TE = 30 ms), field of view (FOV = 224×224 mm), matrix size (64×64), and 17 slices (thickness = 4 mm, 1 mm gap), resulting in a voxel size of 3.5×3.5×5 mm^3^, ensuring full coverage of the visual, parietal and motor cortices with limited coverage of prefrontal cortex and the cerebellum. Anatomical images were collected with a sequence based on the Alzheimer’s Disease Neuroimaging Initiative (ADNI) (parameters: TR = 2250 ms, TE = 2.6 ms, FA = 9°, FOV 256×256 mm^2^, 256×256 matrix, 192 slices, slice thickness = 1 mm, duration = 8∶26 min).

### Behavioral Data Analysis

The behavioral data were analyzed using custom code in MATLAB (R2010a; The MATHWORKS Inc., Natick, MA, USA) and SPSS Statistics (PASW Statistics 18; IBM Corporation, Armonk, NY, USA). As the participants’ instruction was to perform the required order of the left and right button presses as accurately as possible, the following types of errors were defined: a) *unimanual* tapping: a button press with the opposite index finger, b) *bimanual synchronous* and *bimanual unbalanced* tapping: a button press that was delayed more than 100 ms in reference to the button press of the opposite index finger, c) *bimanual alternating* tapping: each additional consecutive button press. Secondly, the actual individual tapping speed during each of the 128 task blocks was estimated by computing the inter-response intervals between the responses made with the right index finger. Both sets of behavioral measures were analyzed using a general linear model with linear contrasts to test for a linear increase along the dimensions of speed and demand on bimanual coordination. Statistical tests for an increase on demand of bimanual coordination were performed across all tapping sequences, as well as across the three bimanual sequences (testing for a linear increase from bimanual *synchronous* to *alternating* to *unbalanced)*. All tests were performed on group-level (fixed effects, the individual block measures were concatenated into one data set), as well as on single-subject level.

### MRI Data Analysis

Functional and anatomical images were pre-processed and analyzed using BrainVoyager QX (Version 2.3, Brain Innovation B.V., Maastricht, The Netherlands), custom code in MATLAB, and SPSS Statistics. The first five volumes of each functional run were discarded due to T1 saturation effects. The data was pre-processed using interscan slice-time correction, 3D rigid-body motion correction, and temporal high-pass filtering with a general linear model (GLM) Fourier basis set, and up-sampled to 3×3×3 mm^3^. Noise artifacts were removed from the data using a linear regression approach. Estimated head motion parameters (three translational, three rotational) to model motion artifacts [28], [30], a localized estimate of the white matter signal to model scanner artifacts [29], and the ventricular signal to model physiological artifacts, were included in the noise model [26], [27]. This combination of nuisance regressors has been recommended to be efficient in increasing the specificity [27], [28], [30], as well as the reliability of the results in functional connectivity analysis [31]. All anatomical and functional data were spatially normalized to Talairach space to enable a comparison between participants [43].

### Group Level Functional Network Analysis

**Figure 2:**
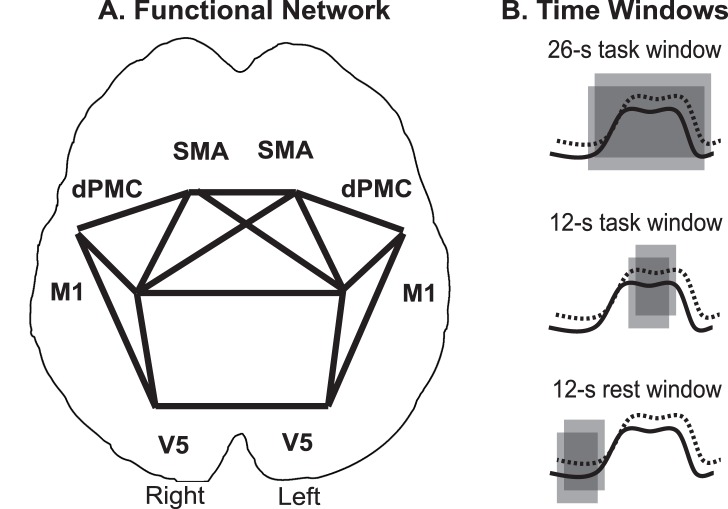
Schematic representation of functional network. All fMRI-based measures were derived from a network of a priori selected regions of interest (M1 = primary motor cortex, dPMC = dorsal premotor cortex, SMA = supplementary motor area, V5 = visual motion area), which are depicted schematically in panel **A**. The time windows (grey boxes) used in the functional connectivity analysis are superimposed on the schematic BOLD responses of the two regions of interest (solid and dotted line) in panel **B**.

For the functional network analysis, eight a priori regions of interest (ROIs) were selected based on the reviewed literature and a meta-analysis on finger tapping [44]; left and right primary motor cortex (M1), left and right supplementary motor area (SMA), left and right dorsal premotor cortex (dPMC), left and right visual motion area (V5) (**figure 2A**). All ROIs were defined individually for each participant, based on the data from the localization run, by computing a GLM with the nuisance predictors and task predictors convolved with a standard two-gamma hemodynamic response function. The ROIs were defined by selecting the 20 most significant functional voxels from the activation cluster closest to the respective coordinates reported in the meta-analysis [44]. In a second step the average time courses of these ROIs were extracted from the pre-processed and spatially normalized data from the experimental runs. Pearson’s correlation coefficients of the activation-level changes in the selected ROIs were computed block-wise for the 128 experimental task blocks of each individual. Three different sets of correlations were calculated using three different time windows: 1) a wider task window that included task on- and offset responses to measure the overall task connectivity (26-s “full task” window, encompassing the rise and decline of the positive BOLD response from 2 seconds after task onset until 28 seconds after, when the decline is expected to level off), 2) a narrow task window omitting task on- and offset responses to compute the steady-state task connectivity during continuous task performance (12-s “steady-state” task window, encompassing only the plateau of the BOLD response from 10 seconds until 22 seconds after task onset, to exclude the initial onset and peak), and 3) a narrow rest window to measure rest connectivity (12-s “rest” window, starting 12s before task onset) (**figure 2B**). Fisher Z transformation was applied to all correlation values to improve the normality of the calculated correlation coefficients [45]. As a preliminary step to the main analysis on single-subject level, we submitted the block-wise correlations to the same group-level statistical analyses (fixed effects) as the behavioral data.

### Within-participant Region-of-interest Analysis

Based on the group-level analysis, the functional connection showing the strongest task modulation was selected in order to further investigate the feasibility of using functional connectivity measures as a neurofeedback measure on a single-subject level. Visual inspection of the single-block blood oxygenation level-dependent (BOLD) responses from the selected ROIs confirmed that the steady-state task- and rest-connectivity (12-s steady-state task window and 12-s rest window) were not contaminated by the task on- and offset responses. For the block-wise correlations the same statistical analyses as performed at the group-level were repeated at the single-subject level. Additionally, block-wise activation level measures were computed for all 128 task blocks (% signal change 12-s task vs. 12-s rest), and submitted to the same statistical analysis. Furthermore, we investigated two different types of brain-behavior link. First, to evaluate the sensitivity and specificity of the computed brain measures to detect if a task was performed uni- or bimanually, we set the intermediate value between the means from both types of tasks as a threshold. We then computed how well the actually performed task could be detected based on the single-block brain measures using this simple threshold approach (chance level being 50%). We tested for significance through paired t-tests. Second, to estimate the criterion validity of the brain measures for indicating which tapping speed was performed, we correlated the block-wise brain measures with the block-wise actual finger tapping speed. We tested for the significance of this correlation through linear regression. Finally, to directly test for differences between the different sets of functional connectivity and activation level-based measures statistically, we submitted them pairwise to a three-way (measure × tapping speed × demand on bimanual coordination) analysis of variance (ANOVA) for repeated measures on group (fixed effects) and single-subject level.

## Results

### Behavioral Data

**Figure 3:**
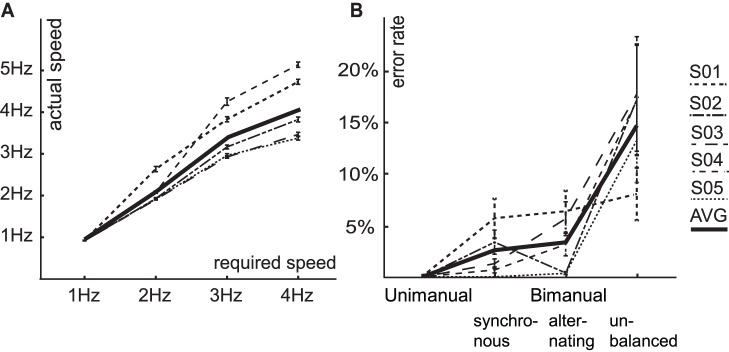
Behavioral results. The behavioral results showed a significant linear increase of tapping speed consistent with the experimental manipulation for all participants. The actual tapping speed of the right index finger from all individuals (S01-05, mean ± individual SE), as well as the average (AVG) is plotted dependent on the required speed in panel **A**. Second, there was a significant linear increase of error rate with increasing demand on bimanual coordination (from left to right) in all participants. In panel **B** the individual error rate (S01-05, mean ± individual SE), and average (AVG) is plotted for the four performed tapping sequences.

All participants completed the session as intended. The linear modulation of actual tapping speed of the right index finger was highly significant in all participants (Group: F(1,39) = 748, p<0.001, Single Subject: F(1,7) >2850, p<0.0001), indicating that there was a linear increase in performed tapping speed as required (**figure 3A**). Also, as expected, a significant linear increase in error rate was found in all participants when demand on bimanual coordination increased across the four different types of tapping sequences (Group: F(1,39) = 1997, p<0.001, Single Subject: F(1,7) >545, p<0.0001), or across the three bimanual tapping sequences (Group: F(1,39) = 1335, p<0.001, Single Subject: F(1,7) >239, p<0.0001) (**figure 3B**). The modulation of error rate by tapping speed was not linear. In four participants, the results showed a significant quadratic effect of speed, with most errors being made during the slowest and the fastest conditions (Group: F(1,39) = 7.9, Single Subject: F(1,7) >9.2, p<0.019). Finally, the behavioral data from these four participants showed a significant interaction effect between demand on bimanual coordination and actual tapping speed (Group: F(1,39) = 6.0, Single Subject: F(1,7) >29.2, p<0.001), with a stronger modulation of error rate by demand on bimanual coordination when the tapping speed was higher.

### Group-level Functional Network

**Figure 4:**
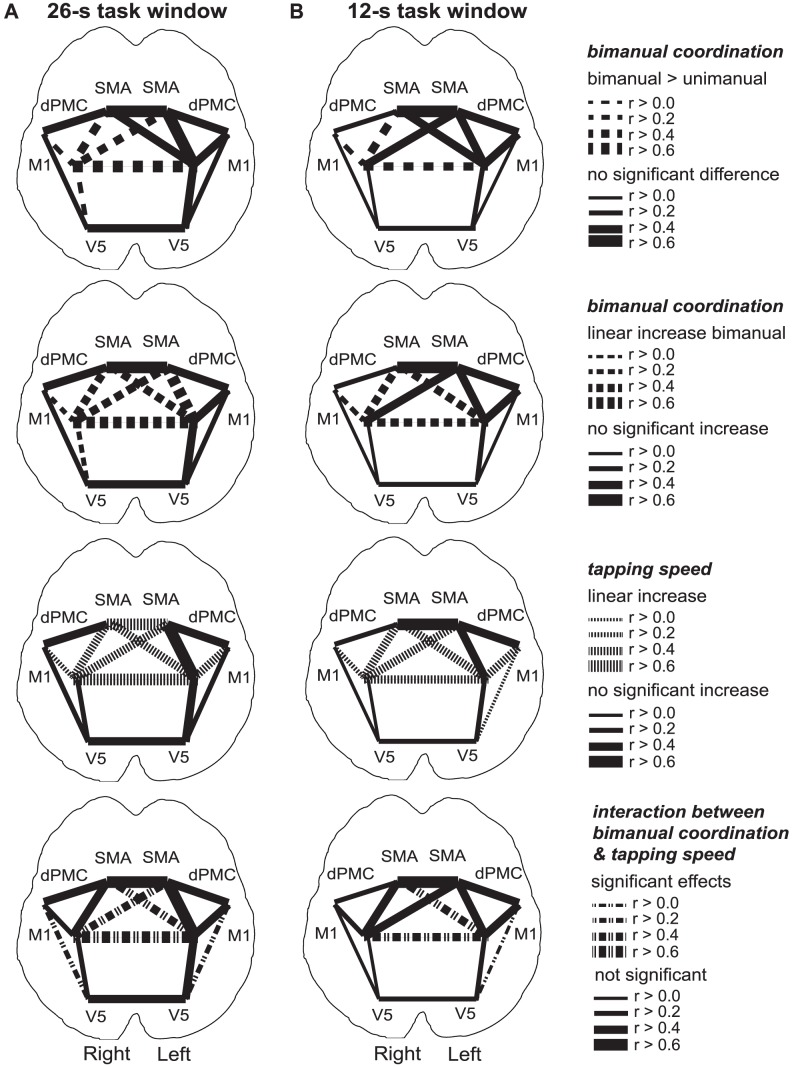
Group-level functional network results. The task-dependent modulation of the group-level overall task connectivity (26-s full task window, depicted on the left) and the steady-state task connectivity (12-s steady-state task window, shown on the right) are visualized schematically for the investigated functional network (M1 = primary motor cortex, dPMC = dorsal premotor cortex, SMA = supplementary motor area, V5 = visual motion area). The upper row shows the significant difference in functional connectivity between unimanual and bimanual tapping. The second row depicts the significant linear increase of functional connection with increasing demand on bimanual coordination during bimanual tapping. The third row shows how connectivity significantly increased with increasing tapping speed. The bottom row depicts the significant interaction effects between demand on bimanual coordination and tapping speed. While the effects were weaker during steady-state in comparison to overall task connectivity, the task-dependent modulations were qualitatively very similar independent of the time window used. Only one connection, the connection between the two primary motor cortices, showed all effects independent of the time window used. This connection also showed the highest average correlation in the functional network (the thickness of the depicted connections equals the average correlation across all experimental conditions).

In all participants the eight ROIs were functionally localized based on the independent data from the localization run (**table 1**). In the functional network derived from the rest periods immediately preceding each task block (12-s rest window) only one of the fifteen analyzed connections was significantly linearly modulated by tapping speed in the preceding task period (left-right SMA: F(1,39) = 8.2, p = 0.007, not depicted), and demand on bimanual coordination (left-right SMA: F(1,39) = 9.5, p = 0.004, not depicted). In the analysis of the functional connectivity networks derived from the two task windows (26-s full task and 12-s steady-state task window), a number of connections showed a significant increase of functional connectivity with increasing tapping speed (e.g., left-right M1 26-s full task window: F(1,39) = 26.6, p<0.001, 12-s steady-state task window: F(1,39) = 6.7, p = 0.01), and increasing demand on bimanual coordination (e.g., left-right M1 unimanual vs. bimanual 26-s full task window: F(1,39) = 523.5, p<0.0001, 12-s steady-state task window: F(1,39) = 18.5, p<0.001; left-right M1 linear modulation 26-s full task window: F(1,39) = 462.0, p<0.0001, 12-s steady-state task window: F(1,39) = 28.6, p<0.001) (**figure 4A** **,** **3B**). In general, the task effects in the steady-state task functional network (12 s task window) were weaker but not qualitatively different from the effects found in the overall task connectivity functional network (26 s task window) (**figure 4A** **,** **3B**). Finally, three connections of the functional networks derived from the task data showed significant interaction effects for both time windows (left M1 with right M1, left M1 with right SMA, and left V5 with left dPMC). For these three connections the functional connectivity was highest when both tapping speed and demand on bimanual coordination increased (e.g., left-right M1 26-s full task window: F(1,39) = 9.0, p = 0.005, 12-s steady-state task window: F(1,39) = 5.3, p = 0.027). A comparison between all functional connections showed that the task-dependent effects were strongest for the left-right M1 connection. Further analyses were therefore restricted to this functional connection.

**Table 1:** Individual regions-of-interest.

	left M1			right M1			left SMA			right SMA			left dPMC			right dPMC			left V5			right V5		
	x	y	z	x	y	z	x	y	z	x	y	z	x	y	z	x	y	z	x	y	z	x	y	z
Participant 01	−39	−24	48	40	−22	46	−8	−12	52	3	−10	57	−49	−13	44	50	−9	47	−46	−63	3	40	−62	4
Participant 02	−35	−18	54	33	−22	50	−6	−8	56	1	−2	60	−48	−10	49	42	−5	45	−55	−61	2	37	−66	−1
Participant 03	−37	−17	51	35	−17	56	−7	1	47	5	10	43	−36	−1	55	35	−3	52	−42	−73	2	49	−67	0
Participant 04	−37	−15	58	37	−12	50	−5	−6	51	3	−3	51	−44	−4	51	47	1	52	−44	−75	−6	45	−73	−4
Participant 05	−41	−13	51	35	−18	50	−6	0	54	7	0	53	−47	−6	53	43	−5	48	−45	−63	5	43	−69	5
Group	−38	−17	52	36	−18	50	−6	−5	52	4	−1	53	−45	−7	50	43	−4	49	−46	−67	1	43	−67	1
Meta-analysis	−38	−26	50	36	−22	54	−4	−8	52				47	1	50				−42	−66	4	44	−66	−2

The Talairach coordinates of the functionally defined regions of interest are listed for each individual participant. The Talairach coordinates for the group average and coordinates reported by a meta-analysis of 38 finger tapping studies [44] are shown for comparison. M1 = primary motor cortex, SMA = supplementary motor area, dPMC = dorsal premotor cortex, V5 = visual motion area.

### Within-participant Region-of-interest

**Figure 5:**
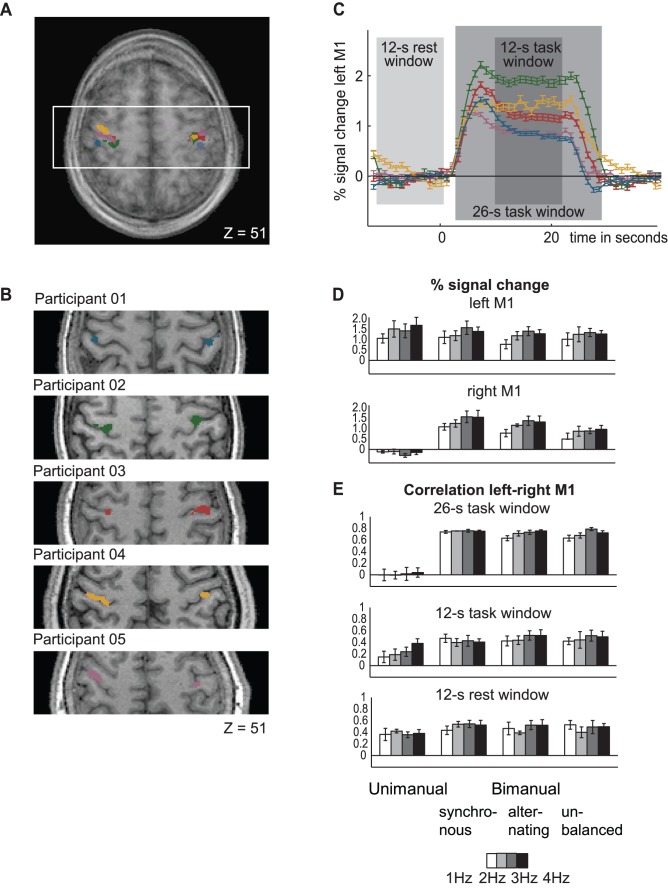
Within-participant region-of-interest results. The individually selected regions of interest in the left and right primary motor cortices (M1) of the five participants are projected onto an average of all participants’ anatomical brain images in panel **A** (z = 51, Talairach space), and onto the individual anatomical brain images in panel **B**. In Panel **C** the BOLD responses from left M1 (averaged across all tasks) are depicted for all participants (mean ± individual SE). The time windows used to compute the block-wise correlations are superimposed on the BOLD responses. Panel **D** displays the average activation level (group mean ± group SE) during each of the sixteen experimental conditions (four different tapping sequences performed at four different speeds) in right and left M1 (group mean ± group SE), while panel E shows the results (group mean ± group SE) from the correlation analysis of the same regions of interest. From unimanual to bimanual finger tapping the average activation level increased, as expected, in the right, but not left primary motor cortex (left M1: *unimanual* 1.4%, *synchronous* 1.3%, *alternating* 1.2%, *unbalanced* 1.2%; right M1: *unimanual* −0.2%, *synchronous* 1.3%, *alternating* 1.2%, *unbalanced* 0.9%). This effect was reflected in the steady-state task and overall task connectivity (26-s full task window: *unimanual*: 0.02, *synchronous* 0.75, *alternating* 0.73, *unbalanced* 0.73; 12-s steady-state task window: *unimanual*: 0.24, *synchronous* 0.42 *alternating* 0.47, *unbalanced* 0.47,), but not visible during rest connectivity (*unimanual* 0.40, *synchronous* 0.49 *alternating* 0.48, *unbalanced* 0.48). Additionally, all task derived measures were modulated by finger tapping speed. For the activation level derived measures, this effect was most pronounced when the performed tapping sequence was easy. During steady-state connectivity the modulation by finger tapping was strongest during *unimanual*, *alternating* and *unbalanced* tapping, and for the overall task connectivity the modulation by finger tapping speed was most pronounced as tapping sequences became most difficult (*alternating* and *unbalanced* tapping).

**Figure 6:**
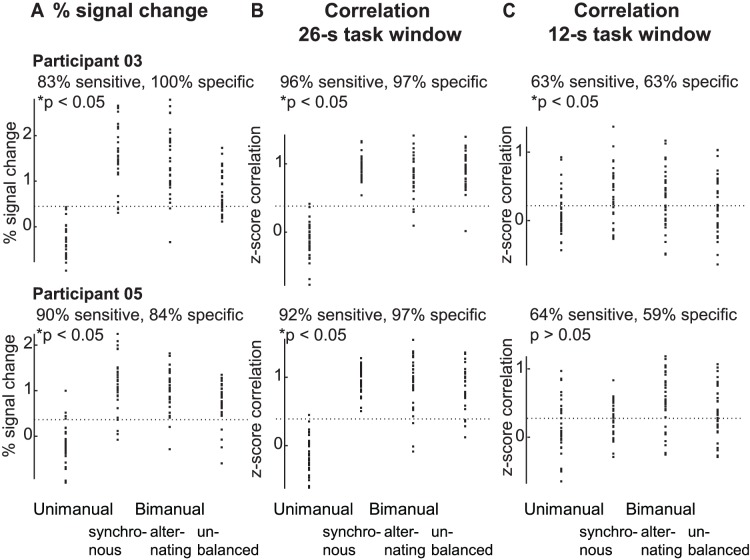
Sensitivity and specificity in detecting bimanual tapping. The sensitivity and specificity in detecting if a task was uni- or bimanually performed was computed using a simple threshold approach. The results for the block-wise activation-level measures from right M1 (panel **A**), and the 26-s full task correlations (panel **B**), and the 12-s steady-state task correlations (panel **C**) are presented for two participants. Each dot represents one block. Significant results are marked with an asterisk. Activation level based and overall task connectivity measures both performed well in making this binary decision, while steady-state connectivity measures performed more poorly, but still above chance level (50%) in three of five participants (see table 2).

**Figure 7:**
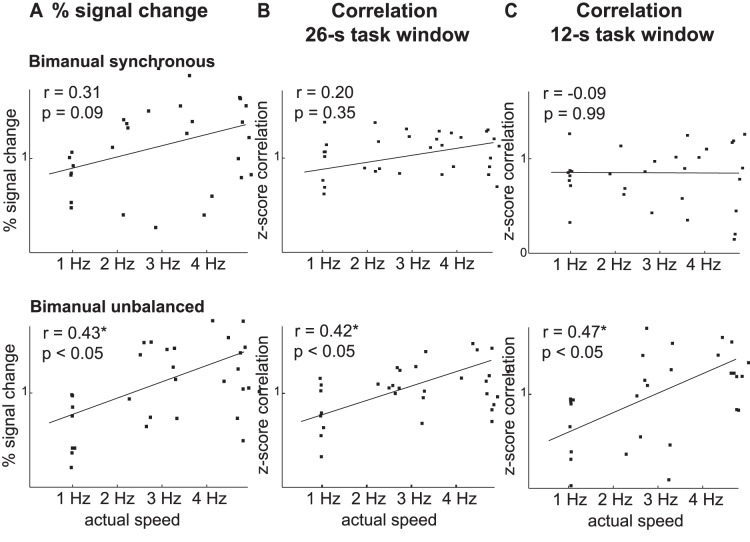
Correlation with finger tapping speed. The criterion validity for detecting performed tapping speed was calculated by correlating the block-wise brain measures with the block-wise behavioral performance measures. The results from one representative participant are depicted for *bimanual synchronous* tapping (upper row) and *bimanual unbalanced* tapping (lower row). The correlation between finger tapping speed and the block-wise activation-level measures from left M1 (panel **A**), the 26-s full task block-wise correlations (panel **B**), and the 12-s steady-state task correlations (panel **C**) are shown. Each dot represents one block, with the regression line indicating the average strength of the brain-behavior correlation. Significant results are marked with an asterisk. The steady-state connectivity measures were modulated by finger tapping speed during the most difficult *unbalanced* tapping task, but not during the easier *synchronous* tapping task. The same effect is visible but less pronounced for the overall task connectivity measures, and much weaker for the activation-level based measures. The connectivity measures thus indicate overall task difficulty best, showing the strongest increase from low to high overall task difficulty.

Figure 5 depicts the individually localized left and right primary motor cortex, the measured BOLD responses from these regions with the functional connectivity analysis time windows superimposed, as well as the group-average activation-level and correlation values derived from this data. The statistical analysis of the individual block-wise activation level measures showed that there was a slight decrease in activation level in the left primary motor cortex with increasing demand on bimanual coordination in four out of five participants (Group: F(1,39) = 14.6, p<0.001, Single Subject: F(1,7) >6.1, p<0.04). Furthermore, as expected, the activation level of this region linearly increased when tapping speed increased, reaching significance in four participants (Group: F(1,39) = 37.2, p<0.001, Single Subject: F(1,7) >7.6, p<0.03; **figure 5D**). There was no significant interaction effect except in one participant (Group: F(1,39) = 1.9, p = 0.17, Single Subject: F(1,7) = 7.0, p = 0.03). In the right primary motor cortex, as expected, there was a strong increase in activation level between unimanual and bimanual tapping (Group: F(1,39) = 297, p<0.001, Single Subject: F(1,7) >86, p<0.001, **figure 5D** **,** **6A**), but activation levels decreased with increasing demand on bimanual coordination across the three bimanual tasks in four participants (Group: F(1,39) = 297, p<0.001, Single Subject: F(1,7) = p<0.001, **figure 5D**). In both regions of interest the highest activation levels were thus found when finger tapping speed was high and an easier tapping sequence was performed. The interaction effect in the right primary motor cortex was significant in one participant (Group: F(1,39) = 8.7, p = 0.005, Single: Subject: F(1,7) = 23.0, p = 0.002). The individual analyses of the functional connectivity measures on single-subject level confirmed the reported group results, showing robust and consistent differences in task connectivity between the unimanual vs. the bimanual tapping sequences (26-s full task window: all participants, F(1,7) >54.4, p<0.001; 12-s steady-state task window: three participants, F(1,7) >5.9, p<0.04, 12-s rest window: one participant, F(1,7) = 20.6, p = 0.003, **table 2** **,** **figure 5E** **,** **6**). The average sensitivity and specificity in detecting if a task was performed uni- or bimanually was high when based on the activation-level measures from the right primary motor cortex (86% sensitivity, 91% specificity), and the overall task connectivity measures (93%, 91%), and moderate when based on the steady-state task connectivity measures (68%, 59%) (**table 2** **,** **figure 6**). Second, as in the group-level analysis, task connectivity also increased when tapping speed increased. This linear effect was significant for both the 26-s full task window (three participants, F(1,7) >6.1, p<0.04) and the 12-s steady-state task window (two participants, F(1,7) >4.9, p<0.002), and non-significant during rest (F(1,7) <1.8, p>0.22). However, the individual functional connectivity analyses showed an interaction effects for four participants with the 26-s full task window (F(1,7) >6.5, p<0.04), and three participants in the steady-state task connectivity analysis (F(1,7) >6.3, p<0.04), being non-significant during rest (F(1,7) <0.4, p>0.55). The modulation of the task connectivity by tapping speed was most pronounced when demand on bimanual coordination was high, and overall task difficulty was therefore increased. This effect was unique to the two sets of task connectivity measures, as the modulation of the activation level-based measures by tapping speed did not become more pronounced with increasing demand on bimanual coordination in both left and right primary motor cortex (figure 5, figure 7). Post hoc tests regarding the linear effect of tapping speed, performed separately for the three bimanual tapping tasks, confirmed this interaction effect (**table 3** **,** **figure 7**). The criterion validity of the correlation measures regarding tapping speed increases from *synchronous* tapping (steady-state = −0.06/overall = 0.09), to *alternating* tapping (steady-state = 0.14/overall = 0.29), to *bimanual unbalanced* tapping (steady-state = 0.14/overall = 0.34), while this effect was not found for the activation level-based measures (*synchronous*: 0.35, *alternating*: 0.36, *unbalanced* 0.30, **table 3** **,** **figure 7**). Overall, the task connectivity measures thus differentiate best regarding overall task difficulty, showing the strongest increase from low to high overall difficulty. A direct statistical comparison between the different sets of measures confirmed that both sets of activation level-based measures differed statistically from both sets of task connectivity measures (26-s full task window vs. right M1: interaction effect measure*bimanual coordination: Group F(3,117) = 22.5, p<0.001, significant in three participants, F(3,21) >9.7, p<0.001; 26-s full task window vs. left M1: interaction effect measure*bimanual coordination: Group F(3,117) = 23.9, p<0.001, significant in four participants, F(3,21) >15.8, p<0.001; 26-s full task window vs. left M1: interaction effect measure*tapping speed: Group F(3,117) = 7.7, p<0.001, significant in four participants, F(3,21) >3.1, p<0.04); 12-s full task window vs. right M1: interaction effect measure*bimanual coordination: Group F(3,117) = 77.5, p<0.001, significant in all participants, F(3,21) >10.7, p<0.001; 12-s full task window vs. left M1: interaction effect measure*bimanual coordination: Group F(3,117) = 14.6, p<0.001, significant in three participants, F(3,21) >15.8, p<0.008; 12-s full task window vs. left M1: interaction effect measure*tapping speed: Group F(3,117) = 7.7, p<0.001, significant in three participants, F(3,21) >5.0, p<0.009)).

**Table 2:** Sensitivity and specificity in detecting bimanual tapping.

	Block activation-levelright M1	Block correlations 26-s fulltask window	Block correlations 12-ssteady-state task window	Block correlations 12-srest window
Participant 01	86%/91%*	94%/88%*	67%/56%	68%/56%
Participant 02	86%/84%*	94%/97%*	79%/66%*	75%/47%*
Participant 03	83%/100%*	96%/97%*	63%/63%*	49%/47%
Participant 04	85%/94%*	88%/75%*	69%/53%*	54%/31%
Participant 05	90%/84%*	92%/97%*	64%/59%	66%/63%
Group	86%/91%	93%/91%	68%/59%	62%/49%

The sensitivity and specificity (sensitivity/specificity) in detecting if a task was performed with only the right index finger (*unimanual*), or with both index fingers (*bimanual*) was computed for all participants using a simple threshold approach (bold with asterisk = significant results).

**Table 3:** Correlation with finger tapping speed.

	Block activation-levelleft M1	Block correlations 26-sfull task window	Block correlations 12-ssteady-state task window	Block correlations 12-srest window
Bimanual synchronous				
Participant 01	0.26	0.26	−0.01	0.15
Participant 02	0.18	0.03	−0.11	0.16
Participant 03	0.61*	0.01	0.11	0.30
Participant 04	0.31	0.20	−0.09	−0.14
Participant 05	0.40*	−0.03	−0.21	−0.18
Group	0.35	0.09	−0.06	0.06
Bimanual alternating				
Participant 01	0.52*	0.24	−0.21	0.30
Participant 02	0.05	0.03	0.07	0.06
Participant 03	0.62*	0.08	0.20	0.17
Participant 04	0.22	0.62*	0.43*	0.06
Participant 05	0.39*	0.47*	0.21	0.04
Group	0.36	0.29	0.14	0.13
Bimanual unbalanced				
Participant 01	0.39*	0.14*	−0.11	0.01
Participant 02	−0.20	0.34	0.15	−0.04
Participant 03	0.74*	0.33*	0.01	−0.01
Participant 04	0.43*	0.42*	0.47*	0.12
Participant 05	0.12	0.46*	0.20	0.04
Group	0.30	0.34	0.14	0.03
Averaged across bimanual tapping tasks				
Participant 01	0.34*	0.18	−0.09	0.16
Participant 02	0.06	0.13	0.00	0.06
Participant 03	0.61*	0.17*	0.09	0.14
Participant 04	0.31*	0.38*	0.25*	0.00
Participant 05	0.30*	0.34*	0.06	−0.02
Group	0.32	0.24	0.06	0.07

The criterion validity for detecting performed tapping speed was calculated by correlating the block-wise brain measures with the block-wise finger tapping speed. These brain-behavior correlations are presented for all participants for each of the three different bimanual tapping tasks separately as well as averaged (bold with asterisk = significant results).

## Discussion

The purpose of this study was to investigate whether windowed functional connectivity measures in comparison with activation level-based measures may be a good indicator of changes in task performance during a well-controlled, simple behavioral (here motor) task. This was assessed in a small number of subjects as a proof-of principle study using variants of a simple bimanual motor task at a single-trial level to investigate the feasibility of windowed correlations as a (potential) neurofeedback signal. We observed four main findings: first, windowed correlations computed based on very short time windows did indeed provide valid information on certain task aspects. Second, the obtained information was unique, as task connectivity measures were more indicative of overall task difficulty than activation level-based measures. Third, the robustness of the steady-state task connectivity measures with the chosen approach was relatively low, and fourth, the task dependent modulation of functional connectivity was spatially focused within the task-relevant network.

Two different sorts of task connectivity measures were investigated: steady-state task connectivity as an index of integration during continuous performance, and overall task connectivity as a compound measure indexing both steady-state performance and gross activation level changes. For both sets of task connectivity measures significant brain-behavior relationships were found, which were unique in comparison with activation-level based measures. While we hypothesized that functional connectivity measures may be more sensitive to bimanual coordination demands, and less to finger tapping speed, we found that they were most indicative of overall task difficulty. While activation-level based measures increased with increasing finger tapping speed, and decreased with increasing demand on bimanual coordination, steady-state task connectivity increased with increasing tapping speed, as well as with increasing demand on bimanual coordination. The highest activation levels were thus found for high speed tapping during an easy tapping sequence, while steady-state connectivity was highest when overall task difficulty was high. The full task window correlations, a compound measure between activation level changes and steady-state, showed a mix of those two effects. In general, the two sorts of task connectivity measures thus indexed overall task difficulty better than activation level-based measures. While these results need to be interpreted with caution due to the small sample size, and do not allow for population inference, they were consistent across participants.

The findings are in line with previous studies showing that increasing demand on bimanual coordination, or tapping speed during finger tapping leads to higher functional connectivity [36], [37]. The presented findings furthermore corroborate previous research showing that an increasing cognitive demand during the performance of working memory tasks also enhances functional connectivity between task-relevant regions [46], [47], [48], [49]. Finally, the presented findings are consistent with previous imaging studies showing that task-dependent enhanced interhemispheric coupling is highest during early stages of motor skill learning, when task difficulty is highest [41]. Three recent studies on motor learning showed that the functional connectivity within the motor network rapidly increased during an initial stage of learning, and then decreased as learning slowed down and performance stabilized [50], [51], [52]. The results from the presented study, as well as from previous studies thus support the idea that functional connectivity measures may be used as an indicator of overall task difficulty during neurofeedback training. The presented study extends previous findings by showing that short-window correlations can capture this task aspect, and seem to be more indicative than activation level-based measures.

A third finding of this study was the low robustness of the steady-state task connectivity measures. While the steady-state correlations seem to have potential for indexing task aspects, which cannot be captured equally well by activation level-based measures, their reliability was considerably lower. Effect sizes were relatively small and the consistency of the results across participants thus compromised. Further research into improving the stability of steady-state connectivity measures thus seems necessary to make a short-window connectivity-based neurofeedback training implementation feasible. To optimize the data quality through dense sampling we used a relatively high temporal resolution in comparison with standard imaging parameters, compromising on spatial coverage in return. At the same time we attempted to maximize the signal to noise ratio through the use of relatively large functional voxels. However, further optimization of the data quality seems crucial in order to provide robust measures. Further future improvements could be achieved by further reduction of spatial coverage, fundamentally improved hardware, advanced imaging sequences [53], [54], new methods for noise reduction [55], or noise removal [56].

Finally, the network analysis performed on the group-level showed that the task-dependent modulation of the functional connectivity was clearly spatially focused within the analyzed task network. Similar results have been reported by other studies, showing that task-dependent modulations of brain connectivity patterns are often spatially quite restricted [57], [58]. Therefore, the use of bivariate pairwise correlations as a measure of task-dependent modulations in functional networks might be a promising approach for a functional connectivity neurofeedback implementation. The advantage of this approach might be that it would allow for further optimization of the imaging sequence parameters by limiting spatial coverage and increasing temporal resolution. More research will be necessary to confirm if this significantly improves the robustness and sensitivity of the functional connectivity measures.

Overall, the presented results thus support the idea that functional connectivity measures may be valuable indicators of task difficulty for neurofeedback based learning. Functional connectivity neurofeedback could provide relevant, and to a certain extent unique information during neurofeedback training. Windowed correlations may serve as an indicator of overall task difficulty on an individual level, indicating how difficult a task is for this individual at this moment in time. Overall, functional connectivity measures may thus add an important estimate regarding the individual learning process in comparison with the activation level-based feedback measures as previously used in neurofeedback patient training studies [59], [60], [61], [62], and in multivariate real-time approaches currently under investigation [63]. Further research into the generalizability of the results to other task paradigms and patient populations thus seems worth pursuing.

### Conclusions

The present study set out to investigate the general feasibility of fMRI connectivity-based neurofeedback. Our results demonstrate that task connectivity seems to provide unique information on task difficulty. If functional connectivity measures can provide a valid index of individual task difficulty during learning, this might be extremely valuable for patients. During training, especially in a patient setup, participants are often encouraged to adopt and employ novel cognitive, behavioral and emotional strategies. An individual index of task difficulty could encourage patients to constantly perform at a high level of individual difficulty, something that may necessary for mastering the novel cognitive, behavioral and emotional skills, which patients are lacking. If the results of this study could be generalized, windowed functional connectivity neurofeedback may therefore indeed become a valuable additional tool for neurofeedback training setups.
